# 183. Impact of Revised Piperacillin/Tazobactam Clinical Breakpoints on Enterobacterales Isolates Identified in Blood Cultures

**DOI:** 10.1093/ofid/ofac492.261

**Published:** 2022-12-15

**Authors:** Nicholas M Moore, Joyce H Houlihan, Christy A Varughese, Hayley A Hodgson, Shivanjali Shankaran, Sarah Y Won, Mary K Hayden

**Affiliations:** Rush University Medical Center, Chicago, Illinois; Rush University Medical Center, Chicago, Illinois; Rush University Medical Center, Chicago, Illinois; Rush University Medical Center, Chicago, Illinois; Rush University Medical Center, Chicago, Illinois; Rush University Medical Center, Chicago, Illinois; Rush University Medical Center, Chicago, Illinois

## Abstract

**Background:**

Enterobacterales are a significant cause of bloodstream infections (BSI) in hospitalized patients. Prior evidence suggests that treatment with piperacillin/tazobactam may not be ideal for BSI. Piperacillin/tazobactam clinical breakpoints were recently updated by the Clinical and Laboratory Standards Institute (CLSI).

**Methods:**

We retrospectively evaluated Enterobacterales blood isolates identified by MALDI-TOF (Vitek MS, bioMérieux) between January 1, 2017 through December 31, 2021 at Rush University Medical Center in Chicago, Illinois. Antimicrobial susceptibility testing was performed using NM43 or NM56 panels on the MicroScan WalkAway 96 (Beckman Coulter). The range of dilutions of piperacillin/tazobactam included on both panels was 8/4 to 64/4 μg/mL. Minimal inhibitory concentrations (MIC) were interpreted using current CLSI breakpoints.

**Results:**

We evaluated 1597 Enterobacterales isolates. Most isolates identified were *Escherichia coli* [n=806 (50%)] and *Klebsiella pneumoniae* [n=358 (22%)]. The majority of isolates (90.4%) were susceptible; 28 (1.8%) isolates were susceptible-dose dependent and 125 (7.8%) were resistant to piperacillin/tazobactam using the new CLSI breakpoints (**Table**). 236 isolates had a multidrug-resistant phenotype, of which 216 (92%) were confirmed as an ESBL. Among ESBL-producing isolates, the majority (81%) were susceptible [MIC ≤ 8 µg/mL) to piperacillin/tazobactam. Isolates with confirmed carbapenemase production had off-scale MICs ( > 64 µg/mL) (**Figure**).

Piperacillin/Tazobactam Breakpoint Revision Table

**Figure:**
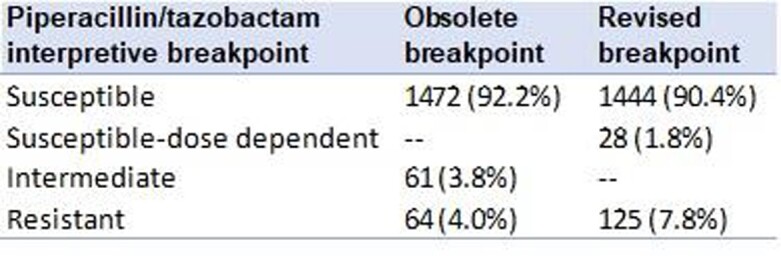

Overall changes in piperacillin/tazobactam interpretations using revised breakpoints.

MDR-Enterobacterales piperacillin/tazobactam MIC distributions

**Figure:**
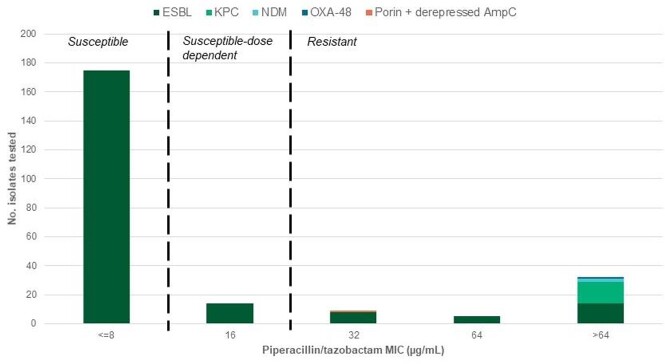

Piperacillin/tazobactam minimal inhibitory concentration distributions among multidrug-resistant Enterobacterales isolates.

**Conclusion:**

While there was an increase in resistance, our results indicate that most Enterobacterales isolates tested susceptible to piperacillin/tazobactam using the revised CLSI breakpoints.

**Disclosures:**

**Nicholas M. Moore, PhD, D(ABMM)**, Abbott Molecular: Grant/Research Support|Cepheid: Grant/Research Support.

